# Phylogenetic analysis and prevalence of Delta hepatitis among HBsAg carriers in Afghanistan

**DOI:** 10.22099/mbrc.2022.44692.1780

**Published:** 2022

**Authors:** Abbas Ali Husseini, Mehran Rostamzadeh

**Affiliations:** Life Science, and Biomedical Engineering Application and Research Center, Istanbul Gelisim University, Istanbul 34310, Turkey

**Keywords:** Hepatitis Delta, genotype, Phylogenetic analysis, Afghanistan

## Abstract

The molecular profile of hepatitis Delta in Afghanistan remains unclear yet, therefore this study addresses the genotype of HDV among HBsAg carriers in Afghanistan. In total 234 HBsAg-positive sera were examined by chemiluminescent micro-particle immunoassay to detect Anti-HDV antibodies. Serologically positive samples were later approved via real-time PCR test and subsequently, a 731 bp segment of the HDV Delta antigen RNA region was sequenced in the Illumina platform. The isolates were genotyped via distance matrix/UPGMA analysis using Kimura 2-parameter by MEGA7 software package program. The HBV/HDV coinfection rate among HBsAg carriers in Afghanistan was 2.1%. Finally, 4 samples successfully amplified Hepatitis delta antigen (HDAg) which Later in phylogenetic analysis, all resided in branch genotype I and were stored at GenBank with accession numbers MK799645, MK799646, MK799647, MK799648. The HDV genotypic variations in the Afghan HBsAg carriers may be homogenous and HDV-1 may be the predominant genotype in Afghanistan.

## INTRODUCTION

Hepatitis delta virus (HDV) is a subviral agent of hepatitis B virus (HBV), and its life cycle is dependent on HBV. Diseases caused by HBV/HDV coinfection are one of the driving open health challenges in low-income countries such as Afghanistan [1]. There are exceptionally few studies on viral hepatitis especially on HDV in this country [2]. 

Seroprevalence and molecular characterization studies on infectious diseases are a necessity to develop proper and adequate public health policies [3, 4]. HBV/HDV coinfection may be a  momentous malady with extreme results depending on the host and viral factors. The variety within the viral nucleotide sequences is one of the key factors that influence the course and  outcomes  of  the diseases [5, 6]. Viral nucleotide sequence varieties in overabundance of 19%-38% in the full genome of HDV characterize the eight HDV (I-VIII) genotypes with ethno – geographical dissemination around the world [7-14].

The impact of genotypes on disease outcomes was examined in many studies. Distinct HBV genotypes are associated with diverse clinical manifestations such as liver disease severity and chronicity, development of hepatocellular carcinoma, response to interferon treatment, and even transmission route and liver transplantation [15]. The results of coinfection with HDV are ordinarily more extreme than HBV contamination alone [16]. The clinical impact of HDV-1 is very variable changing from mild to severe disease. HDV-2 is usually linked to a milder hepatitis, while HDV-3 can lead to fulminant hepatitis mostly. However, there is limited information on the clinical course of the other five HDV genotypes [17].

Molecular epidemiological data are imperative for the development of health strategies. In Afghanistan, the molecular epidemiology of HDV remains definitely uncertain [18]. Due to the lack of such information in Afghanistan, we aimed to assess the current molecular profile of HDV in the general population in this country. 

## MATERIALS AND METHODS

**Site of the study and sampling procedures: **In total 234 HBsAg-positive person sera (143 males and 91 females, age mean 40.4±14.5 years old) which were detected out of randomly collected 5897 samples via point-of-care rapid tests (Standard Diagnostics, Korea/ USA) in previous studies and were later examined by chemiluminescent micro-particle immunoassay (Abbott Laboratories, Illinois, USA) approval test were included to the study.

**Serological tests and viral load measurement: **Anti-HDV antibody screening was performed via chemiluminescent micro-particle immunoassay (Abbott Laboratories, Illinois, USA) among all 234 HBsAg positive samples. Viral nucleic acids were extracted from the patient’s sera using the RTA nucleic acid isolation Kit (RTA, Turkey) and HDV RNA was quantitated using a previously published in-house real-time PCR-based method [19].

**HDV RNA genotyping:** A 731 bp segment of the HDV Delta antigen RNA region was amplified via in-house developed conventional reverse transcription polymerase chain reaction (RT- PCR) and subsequently sequenced in the Illumina platform. The HDV Delta antigen nucleic acid sequences were then used for phylogenetic analysis and genotyping.

**Phylogenetic analysis: **All sequence isolates obtained from HDV-positive sera were manually edited and aligned with corresponding regions of reference sequences retrieved from the GenBank database. The phylogenetic comparison was done by distance matrix/UPGMA analysis using Kimura 2-parameter by MEGA7 software package program (Kumar et al. Institute for Genomics and Evolutionary Medicine, Temple University) [20].

## RESULTS AND DISCUSSION

**Figure 1:**
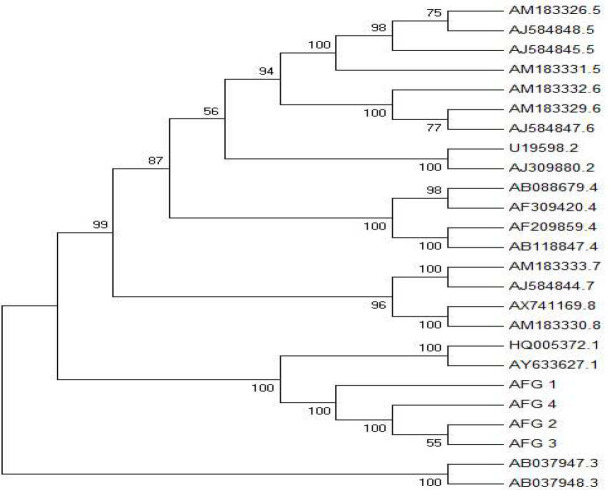
Phylogenetic tree obtained by distance matrix/UPGMA comparison (with Kimura-2correction) after bootstrapping 1000 replicates of sequence segment from HDAg

Out of 234 HBsAg positive samples, the chemiluminescent micro-particle immunoassay displayed co-infection with HDV in 5 individuals with an age mean of 49.2±5 years old. All anti-HDV–positive samples were confirmed by real-time PCR with viral loads ranging from 5.3×103 to 2.2× 104 IU/ml which subsequently 4 samples successfully amplified Hepatitis delta antigen (HDAg). Later in phylogenetic analysis, all resided in branch genotype I (Figure 1) and stored at GenBank with accession numbers MK799645, MK799646, MK799647, MK799648.

This is the first phylogenetic analysis of HDV aiming to determine the genotypes prevalent in the HBsAg carriers in Afghanistan. There was no updated and comprehensive data about HDV molecular epidemiology in Afghanistan. The seroprevalence of HDV infection in central Asia which consider the northern neighbor of Afghanistan was 8.3% in the general population and 51.3% in HBsAg- positive patients [21].

Seroprevalence of HDV among HBV carriers in Pakistan (at least 17%) and Iran (at least 3.9%), the other two major neighboring regions also are relatively high. This study shows that the seroprevalence of HBV/HDV coinfection in Afghanistan among HBV carriers is in line with neighbor regions and is estimated at least 2.1%. 

HDV is classified into HDV-1 to HDV-8 genotypes whose distribution is limited geographically except globally predominant HDV-1 [22]. Among others, Genotypes 2 and 4 are found predominantly in Asia. In Afghanistan, all patients with HDV are infected with genotype 1, which has been associated with the most pathogenic effect among HBV/HDV coinfected patients [21, 22].

## Conflict of Interest:

The authors have no conflict of interest to declare.

## References

[1] Riaz M, Idrees M, Kanwal H, Kabir F (2011). An overview of triple infection with hepatitis B, C and D viruses. Virol J.

[2] Husseini AA, Saeed KMI, Yurdcu E, Sertoz R, Bozdayi AM (2019). Epidemiology of blood-borne viral infections in Afghanistan. Arch Virol.

[3] Toy M, Onder FO, Idilman R, Idilman R, Kabacam G, Richardus JH, Bozdayi M, Akdogan M, Kuloglu Z, Kansu A, Schalm S, Yurdaydin C (2012). The cost-effectiveness of treating chronic hepatitis B patients in a median endemic and middle-income country. Eur J Health Econ.

[4] Toy M, Önder FO, Wörmann T, Bozdayi AM, Schalm SW, Borsboom GJ, Rosmalen JV, Richardus JH, Yurdaydin C (2011). Age- and region-specific hepatitis B prevalence in Turkey estimated using generalized linear mixed models: a systematic review. BMC Infect Dis.

[5] Huang Y, Lok AS (2011). Viral factors and outcomes of chronic HBV infection. Am J Gastroenterol.

[6] Yurdaydin C, Idilman R, Bozkaya H, Bozdayi AM (2010). Natural history and treatment of chronic delta hepatitis. J Viral Hepat.

[7] Bozdayi AM, Aslan N, Bozdayi G, Türkyilmaz AR, Sengezer T, Wend U, Erkan O, Aydemir F, Zakirhodjaev S, Orucov S, Bozkaya H, Gerlich W, Karayalçin S, Yurdaydin C, Uzunalimoğlu O (2004). Molecular epidemiology of hepatitis B, C and D viruses in Turkish patients. Arch Virol.

[8] Zein NN (2000). Clinical significance of hepatitis C virus genotypes. Clin Microbiol Rev.

[9] Bukh J, Purcell RH, Miller RH (1994). Sequence analysis of the core gene of 14 hepatitis C virus genotypes. Proc Natl Acad Sci U S A.

[10] Robertson B, Myers G, Howard C, Brettin T, Bukh J, Gaschen B, Gojobori T, Maertens G, Mizokami M, Nainan O, Netesov, Shin T, Simmonds P, Smith D, Stuyver L, Weiner A (1998). Classification, nomenclature, and database development for hepatitis C virus (HCV) and related viruses: proposals for standardization. International committee on virus taxonomy. Arch Virol.

[11] Stuyver L, Arnhem W, Wyseur A, Hernandez F, Delaporte E, Maertens G (1994). Classification of hepatitis C viruses based on phylogenetic analysis of envelope 1 and nonstructural 5b regions and identification of five additional subtypes. Proc Natl Acad Sci USA.

[12] Pascarella S, Negro F (2011). Hepatitis D virus: an update. Liver Int.

[13] Wu JC, Huang IA, Huang YH, Chen JY, Sheen IJ (1999). Mixed genotypes infection with hepatitis D virus. J Med Virol.

[14] Sagnelli E, Alessio L, Sagnelli C, Gualdieri L, Pisaturo M, Minichini C, Di caprio G, Starace M, Onorato L, Macera M, Scotto G, Coppola N (2017). Hepatitis B virus genotypes, epidemiological characteristics, and clinical presentation of HBV chronic infection in immigrant populations living in southern Italy. Hepat Mon.

[15] Guirgis BSS, Abbas RO, Azzazy HME (2010). Hepatitis B virus genotyping: current methods and clinical implications. Int J Infect Dis.

[16] Heidrich B, Serrano BC, Idilman R, Kabacam G, Bremer B, Raupach R, Onder FO, Deterding K, Zacher BJ, Taranta A, Bozkaya H, Zachou K, Tillmann HL, Bozdayi AM, Manns MP, Yurdaydin C, Wedemeyer H (2012). HBeAg-positive hepatitis delta: Virological patterns and clinical long-term outcome. Liver Int.

[17] Opaleye OO, Japhet OM, Adewumi OM, Adewumi OM, Omoruyi EC, Akanbi OA, Oluremi AS, Wang B, Tong HV, Velavan TP, Bock CT (2016). Molecular epidemiology of hepatitis D virus circulating in Southwestern Nigeria. Virol J.

[18] Jacobson IM, Dienstag JL, Werner BG, Brettler DB, Levine PH, Mushahwar IK (1985). Epidemiology and clinical impact of hepatitis D virus (delta) infection. Hepatology.

[19] Karatayli E, Altunoğlu YT, Karatayli SC, Alagoz SG, Cinar K, Yalcin K, Idilman R, Yurdaydin C, Bozdayi AM (2014). A one step real time PCR method for the quantification of hepatitis delta virus RNA using an external armored RNA standard and intrinsic internal control. J Clin Virol.

[20] Kumar S, Stecher G, Tamura K (2016). MEGA7: Molecular evolutionary genetics analysis version 7 0 for bigger datasets. Mol Biol Evol.

[21] Hale Gokcan, Idilman R (2021). Hepatitis D infection in Asia: A perspective from an endemic region. Clin Liver Dis (Hoboken).

[22] Karlsen AA, Kyuregyan KK, Isaeva OV, Kichatova VS, Asadi Mobarkhan FA, Bezuglova LV, Netesova IG, Manuylov VA, Pochtovyi AA, Gushchin VA, Sleptsova SS, Ignateva ME, Mikhailov MI (2022). Different evolutionary dynamics of hepatitis B virus genotypes A and D, and hepatitis D virus genotypes 1 and 2 in an endemic area of Yakutia, Russia. BMC Infect Dis.

